# Structural analysis of tumor-related single amino acid mutations in human MxA protein

**DOI:** 10.1186/s40880-015-0055-1

**Published:** 2015-09-28

**Authors:** Jia-Li Hu, Yi-Jun Hua, Yang Chen, Bing Yu, Song Gao

**Affiliations:** Sun Yat-sen University Cancer Center, State Key Laboratory of Oncology in South China, Collaborative Innovation Center for Cancer Medicine, Guangzhou, Guangdong 510060 P.R. China

**Keywords:** Human cancer, MxA protein, Single-point mutation, Crystal structure, Domain distribution, Protein stability, Protein folding, Residue interaction, Stereochemistry, Polarity

## Abstract

**Background:**

Human myxovirus resistant protein A (MxA), encoded by the myxovirus resistance 1 (*Mx1*) gene, is an interferon (IFN)-triggered dynamin-like multi-domain GTPase involved in innate immune responses against viral infections. Recent studies suggest that MxA is associated with several human cancers and may be a tumor suppressor and a promising biomarker for IFN therapy. *Mx1* gene mutations in the coding region for MxA have been discovered in many types of cancer, suggesting potential biological associations between mutations in MxA protein and corresponding cancers. In this study, we performed a systematic analysis based on the crystal structures of MxA and elucidated how these mutations specifically affect the structure and therefore the function of MxA protein.

**Methods:**

Cancer-associated *Mx1* mutations were collected and screened from the COSMIC database. Twenty-two unique mutations that cause single amino acid alterations in the MxA protein were chosen for the analysis. Amino acid sequence alignment was performed using Clustal W to check the conservation level of mutation sites in Mx proteins and dynamins. Structural analysis of the mutants was carried out with Coot. Structural models of selected mutants were generated by the SWISS-MODEL server for comparison with the corresponding non-mutated structures. All structural figures were generated using PyMOL.

**Results:**

We analyzed the conservation level of the single-point mutation sites and mapped them on different domains of MxA. Through individual structural analysis, we found that some mutations severely affect the stability and function of MxA either by disrupting the intra-/inter-molecular interactions supported by the original residues or by incurring unfavorable configuration alterations, whereas other mutations lead to gentle or no interference to the protein stability and function because of positions or polarity features. The potential clinical value of the mutations that lead to drastic influence on MxA protein is also assessed.

**Conclusions:**

Among all of the reported tumor-associated single-point mutations, seven of them notably affect the structure and function of MxA and therefore deserve more attention with respect to potential clinical applications. Our research provides an example for systematic analysis and consequence evaluation of single-point mutations on a given cancer-related protein.

## Background

The myxovirus resistance 1 (*Mx1*) gene is one of the most prominent interferon (IFN)-stimulated genes in vertebrate that are highly activated when triggered by type I and III IFNs upon viral infection. Human *Mx1* is located on chromosome 21 and encodes the myxovirus resistant protein A (MxA) protein, which is a major antiviral factor against a wide spectrum of RNA viruses, such as influenza virus and Thogotovirus [1, 2]. The 78-kDa MxA protein is a member of the dynamin superfamily of multi-domain GTPases whose function relies on oligomerization and GTP hydrolysis [3]. MxA has an N-terminal GTPase (G) domain, a stalk region responsible for oligomerization, a bundle-signaling-element domain (BSE) crucial for the communication between the G domain and the stalk region [4–6], and a 40-amino-acid-long loop (conventionally named L4) amid the Stalk on primary structure correlated with the pleckstrin homology (PH) domain in dynamin [3]. Besides, MxA possesses a disordered loop at the N-terminus that is not conserved within the dynamin superfamily. According to the current working model, MxA forms ring-like oligomers around viral ribonucleoparticles (RNPs) and disrupts the structure of RNPs via conformational changes induced by GTP hydrolysis [5].

Apart from its role as a prominent antiviral protein in innate immunity, MxA has been found to be associated with different types of human cancer. It has been reported that the deletion of *Mx1* gene, as a consequence of certain gene fusion events, is closely related to prostate cancer with a high aggressive tendency [7]. Other studies of prostate cancer have suggested that the expression of MxA is suppressed in the highly metastatic human prostate carcinoma cell line PC-3M, and exogenous MxA can inhibit the mobility and invasiveness of PC-3M cells both in vivo and in vitro [8]. Prostate cancer cells in which the *Mx1* gene is knocked out have been shown to be much less sensitive to docetaxel compared with MxA-positive cells [9]. The *Mx1* gene has been found to be hypermethylated and suppressed in primary head and neck squamous cell carcinoma cell lines and tissue samples compared with normal lymphocytes [10]. These studies indicate that *Mx1* is a potential tumor suppressor gene. On the other hand, as a traditional immunotherapeutic reagent [11], type I IFNs are widely used in clinical treatments against a number of human cancers, including renal cell carcinoma, follicular lymphoma, melanoma, and chronic myelogenous leukemia [12]. The effect of type III IFNs against different human cancers, including bladder carcinoma, Burkitt’s lymphoma, colorectal carcinoma, non-small cell lung cancer, and esophageal carcinoma, has also been investigated [13, 14]. As a key cytokine induced by types I and III IFNs, MxA is thought to be a useful biomarker for monitoring IFN activity and predicting clinical efficacy during IFN therapy in patients bearing certain types of cancer [15, 16]. A clinical study on early stage B cell chronic lymphocytic leukemia has suggested that patients with no MxA expression are likely to demonstrate a positive response to IFN therapy [16]. Moreover, the expression level of MxA is also employed to predict the efficacy of chemotherapy on several types of cancer. A worldwide multi-center study has indicated that robust MxA expression is a positive indicator for patients with breast carcinoma who might benefit from anthracycline-based chemotherapy [17].

In recent years, with the development of next-generation sequencing technique and its wide application in cancer studies [18], the landscape of somatic mutations has been revealed for many types of human cancer. According to these comprehensive data sets, *Mx1* mutations have been discovered in a number of common cancer types, including colorectal cancer [19–22], head and neck squamous cell carcinoma [23], follicular lymphoma [24], cutaneous squamous cell carcinoma [25], mantle cell lymphoma [26], embryonal rhabdomyosarcoma [27], renal cell carcinoma [28], prostate cancer [29], lung adenocarcinoma [30], melanoma [31], medulloblastoma [32], and ovarian carcinoma [33]. Given the potential importance of MxA in tumorigenesis and metastasis as well as in the treatment and prognosis of different cancers, additional efforts are needed to investigate the biological associations between *Mx1* mutations, especially the mutations that lead to the malfunction of its encoded protein MxA, and corresponding cancers. Mutations in coding regions can result in different consequences to the translated polypeptides, of which single amino acid alterations take up a considerable portion. These single-point mutations may give rise to various consequences to the function of MxA which, however, are difficult to be predicted and evaluated from the analysis of primary structure. In this study, we exploit the crystal structures of MxA and illustrate how these mutations specifically affect the structure and thus the function of MxA protein.

## Methods

### Data mining

Information on cancer-related mutations in human *Mx1* gene was collected from the Catalogue of Somatic Mutations in Cancer (COSMIC) database [34]. All mutations were manually screened and only the mutations that cause single amino acid alterations in MxA were chosen for subsequent analysis.

### Sequence alignment

Amino acid sequences of *Homo sapiens* (hs) MxA (UniProt code P20591) and MxB (P20592), *Mus musculus* (mm) Mx1 (P09922) and Mx2 (Q9WVP9), *Gallus gallus* (gg) Mx protein (Q90597), *Danio rerio* (dr) MxA protein (Q8JH68), *Homo sapiens* dynamin1 (Q05193), dynamin2 (P50570), and dynamin3 (Q9UQ16), *Drosophila melanogaster* (dm) dynamin (P27619), *Caenorhabditis elegans* (ce) dynamin (Q9U9I9), and *Saccharomyces cerevisiae* (sc) dynamin-related protein DNM1 (P54861) were aligned using Clustal W [35] and manually adjusted for non-conserved loop regions.

### Structural analysis

Crystal structures of full-length MxA (PDB code 3SZR), MxA Stalk (3LJB), apo MxA G domain-BSE fusion construct (Stalkless MxA) (4P4U), and Stalkless MxA complexed with β,γ-methyleneguanosine 5′-triphosphate (GMPPCP, a non-hydrolysable GTP analogue) (4P4S) were used as reference structures. For optimal accuracy, the resolution was considered a prior factor during the selection of reference structures comprising corresponding mutated residues. For mutations within the G domain, the crystal structure of apo Stalkless MxA (1.9 Å resolution) was applied as reference with the exception of L95P and P96S on *Switch I*. For these two mutations, the GMPPCP-bound Stalkless MxA structure (3.3 Å) was taken as reference because only when a GTP analogue is bound to the G domain can *Switch I* be ordered and fully observable. For mutations in the stalk region, the MxA Stalk structure (2.4 Å) was used for structural analysis. For mutations in BSE and Hinge 1, the full-length MxA structure (3.5 Å) was the only choice because other published structural models do not contain these residues. Structural analysis for all mutations was performed using Coot [36]. Interactions among the residues were defined by the linear distance between the centers of the corresponding atoms in the reference models: 2.7–3.5 Å for hydrogen bonds and within 4 Å for salt bridges and hydrophobic interactions. Overall, all reference models provided sufficient information needed for reliable structural analysis.

### Structural modeling of the mutants

Structural models of selected cancer-associated single-point mutants were calculated by the SWISS-MODEL server [37]. In the amino acid sequence input files for each MxA mutant, the original residue was substituted by the post-mutation residue. Mutated MxA sequences were then individually uploaded to the SWISS-MODEL server for model calculation. The templates used for structural model building were assigned according to the corresponding reference models for each mutant as previously mentioned. In the output models, the residues that were absent in the original templates were removed for better quality because these parts may contain severe errors as a result of lacking reference information. The final structural models of the mutants were individually superimposed on the corresponding original reference models using Coot, and the root mean square deviation (r. m. s. d.) values for steric positions of the corresponding atoms between each model pairs were subsequently calculated.

### Structural figure preparation

All structural figures were generated using PyMOL [38]. Different types of atoms in stick or sphere representations were specified by the following colors: red for oxygen, blue for nitrogen, magenta for phosphorous, light green for magnesium, green for sulfur, yellow for carbon in residues that are mutated in human cancers, and gray for carbon in other amino acids that interact with residues of interest in this study. The spheres in the illustration represented Cα of the corresponding residues.

## Results

### Overview of single-point mutations in MxA in different types of cancer

In the COSMIC database, 122 tumor-related mutations were identified in the human *Mx1* gene from whole-genome, whole-exome, and transcriptome sequencing data of human cancers. All 122 mutations reside in coding regions of the MxA protein. For these genetic aberrances, frameshift mutations and termination codon mutations were excluded, as they lead to obvious truncation of the polypeptide chain and therefore the obliteration of MxA structure and function. In addition, silent mutations were also excluded because the amino acid sequence of MxA remains intact in these cases. Consequently, 22 unique cancer-related single amino acid mutations in MxA protein were selected and subjected to subsequent analysis. These 22 MxA mutations, summarized in Table 1, were found across 12 types of human cancer, of which colorectal cancer accounted for half of the mutations, followed by cutaneous squamous cell carcinoma (3 mutations) and mantle cell lymphoma (2 mutations). The other 9 cancers each featured 1 mutation. Two mutations, namely T651M and R655C, both appeared twice. T651M was found in colorectal cancer and head and neck squamous cell carcinoma, whereas R655C was discovered in two separate sequencing projects for colorectal cancer. Overall, MxA single-point mutations are widely spread in different human cancers, and MxA mutates more frequently in colorectal cancer according to current data.

**Table 1:** Summary of reported human myxovirus resistant protein A (MxA) single-point mutations in human cancers

Cancer type	Mutation(s)
Colorectal cancer	S134L, N491K, R522C, T651M, R654Q, V263M, Y538C, S572Y, R655C (2)^a^
Head and neck squamous cell carcinoma	T651M
Follicular lymphoma	T27S
Cutaneous squamous cell carcinoma	L95P, P96S, P218S
Mantle cell lymphoma	G540D, L643V
Embryonal rhabdomyosarcoma	V449G
Renal cell carcinoma	G392V
Prostate cancer	K326N
Lung adenocarcinoma	R310S
Melanoma	E632K
Medulloblastoma	R649W
Ovarian carcinoma	L619I

^a^R655C mutation was discovered in colorectal cancer in two individual sequencing projects

**Fig. 1:**
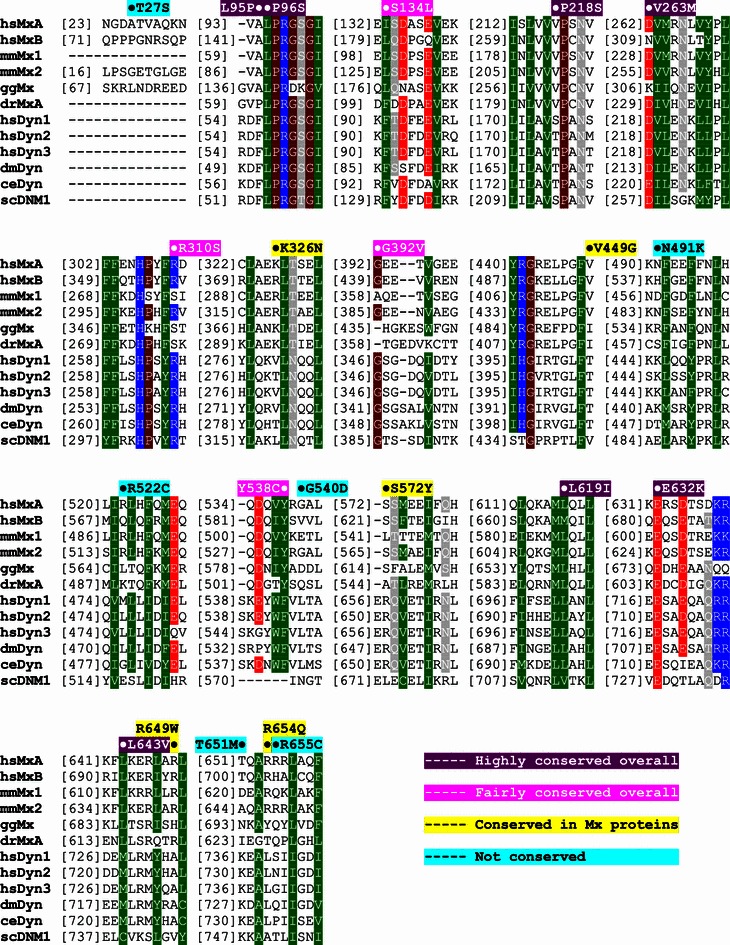
Amino acid sequence alignment of myxovirus resistance (Mx) proteins and dynamins. Amino acid sequences of Mx proteins and dynamins from human, mouse, chicken, zebra fish, fruit fly, *C. elegans*, and yeast are shown in the sequence alignment (see the “Methods” for details). Residues with a conservation of greater than 70% are *color-coded* (negatively charged amino acid residues D and E in *red*; positively charged R, K, and H in *blue*; polar N, Q, S, and T in *grey*; weak or nonpolar A, L, I, V, F, Y, W, M, and C in *green*; and special P and G in *brown*). *Numbers in square brackets* in front of each 10-residue sequence fragment indicate the ordinal position of the first residue of this fragment at the primary structure of the corresponding protein. Tumor-associated mutations are indicated at the corresponding positions. Residues highly conserved in Mx proteins and dynamins (L95, P96, P218, V263, L619, E632, and L643) are shown in *violet*, residues showing considerable overall conservation (S134, R310, G392, and Y538) in *magenta*, residues conserved in Mx proteins but not in dynamins (K326, V449, S572, R649, and R654) in *yellow*, and residues with no conservativeness (T27, N491, R522, G540, T651, and R655) in *cyan*

We then checked the conservation level of the amino acid residues mutated in cancers. Seven residues are highly conserved in Mx proteins and dynamins; 4 residues also show considerable overall conservation. In addition, 5 residues are conserved in Mx proteins but not in dynamins, whereas 6 residues display no conservations at all (Fig. 1). Intriguingly, T651 and R655, although their corresponding mutations appeared more frequently in sequencing data, belong to non-conserved group. This result suggests that it is obscure to infer the physiological importance of these single-point mutations only from their conservativeness in primary structure. To obtain more reliable information, individual analysis of these mutations by structural means is indispensable.

### Distribution of cancer-related single-point mutations in MxA

**Fig. 2:**
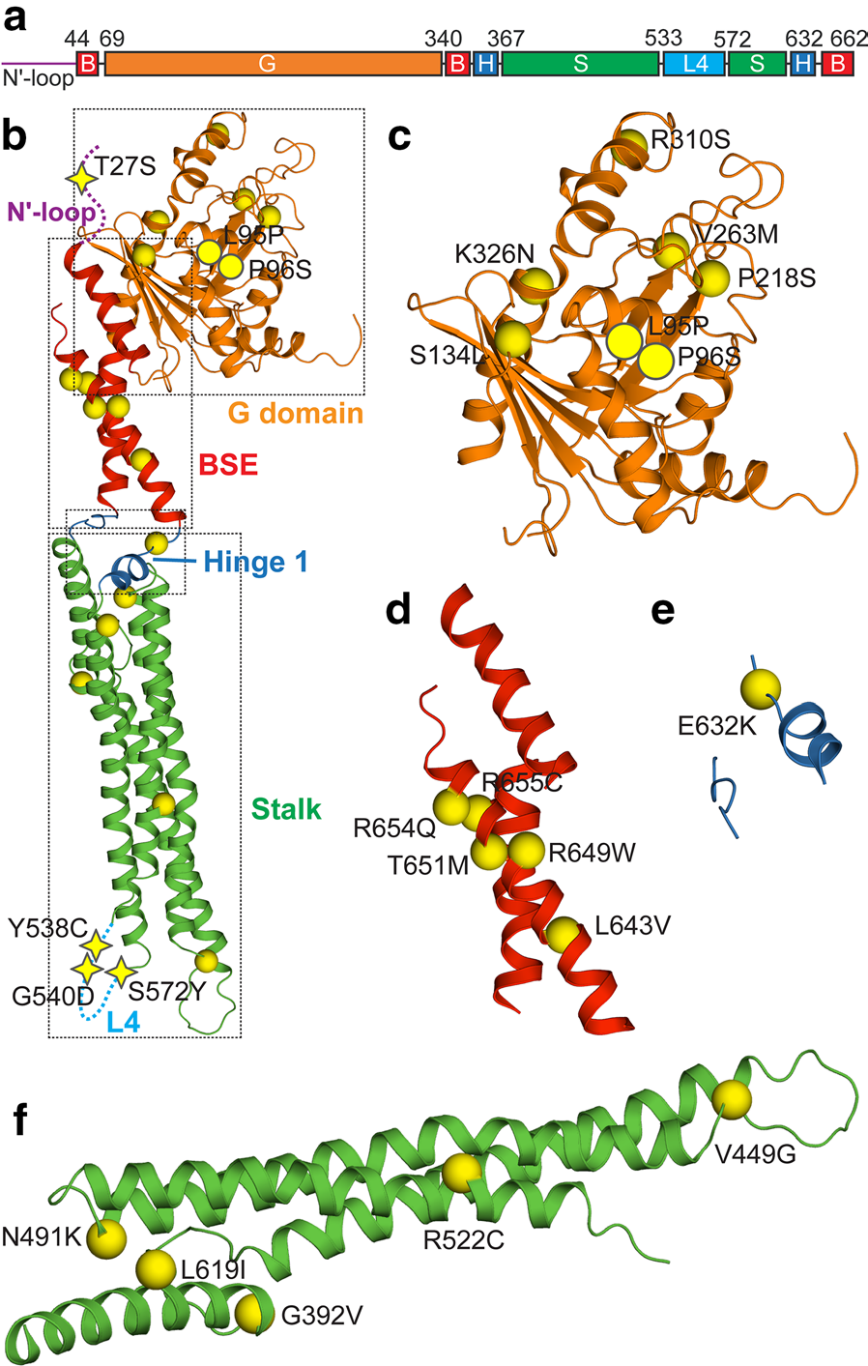
Overview of the distribution of cancer-related mutations within the human myxovirus resistant protein A (MxA) domains. **a** schematic representation of the domain structure of human MxA. *N′-loop* N-terminal disordered loop; *B* bundle-signaling-element domain (BSE), *G* G domain; *H* Hinge 1, *S* Stalk. Borders of the domains are indicated by corresponding residue numbers. **b** overview of the position of all mutations in MxA. The G domain is colored in *orange*, BSE in *red*, Hinge 1 in *sky-blue*, and Stalk in *green*. The missing N-terminal 44 residues (shown in *magenta*) and L4 (shown in *cyan*) are indicated as *dashed lines*. Mutations that are included by the reference model are illustrated as *yellow spheres*. Residues that are missing in this reference model but are present in other reference models are illustrated as *filled yellow circles*. Residues missing in all reference structures are shown as *yellow stars*. **c**–**f** overview of the mutations in individual domains of MxA, as outlined by *dashed rectangles* in Fig. 2a at the corresponding areas: **c** G domain, **d** BSE, **e** Hinge 1, and **f** Stalk. Note that the representations of **e** Hinge 1 and **f** Stalk representations were rotated counter-clockwise 90° from those in Fig. 2b

Human MxA protein is a large dynamin-like GTPase composed of 5 structurally discriminated domains plus an N′-loop (Fig. 2a). Recently, several crystal structures of MxA have been reported, which collectively covered most of the molecule, except for the N′-loop and L4 [4–6]. We took advantage of these structures to perform an in-depth analysis of the 22 cancer-related mutations and their possible structural and functional outcomes on the protein. First, we mapped the mutated residues on MxA molecule and found that these 22 mutations are scattered throughout all regions (Fig. 2b). The G domain, which is largest in size, contains 7 mutation sites, whereas the smaller N′-loop and Hinge 1 each contains 1 mutation site. Considering the size, however, BSE and L4 are relatively more prone to mutation, where 5 and 3 mutation sites are spotted, respectively (Fig. 2c–f). The distribution of the mutations is summarized in Table 2. Next, all single-point mutations were individually investigated according to the domains.

**Table 2:** Domain distribution of cancer-related MxA single-point mutations

MxA segment	Mutation(s) in cancers
GTPase domain (G domain)	L95P, P96S, S134L, P218S, V263M, R310S, K326N
Bundle-signaling-element domain (BSE)	L643V, R649W, T651M, R654Q, R655C
Stalk	G392V, V449G, N491K, R522C, L619I
Hinge 1	E632K
L4	G540D, Y538C, S572Y
N′-loop	T27S

#### Single-point mutations in G domain

The G domain of MxA is responsible for GTP hydrolysis, as well as for the inter-ring homo-dimerization via the nucleotide-binding pocket [39]. Both actions are crucial for the mechano-chemical coupling of the entire oligomer and thus for the function of the protein [5, 6].

**Fig. 3:**
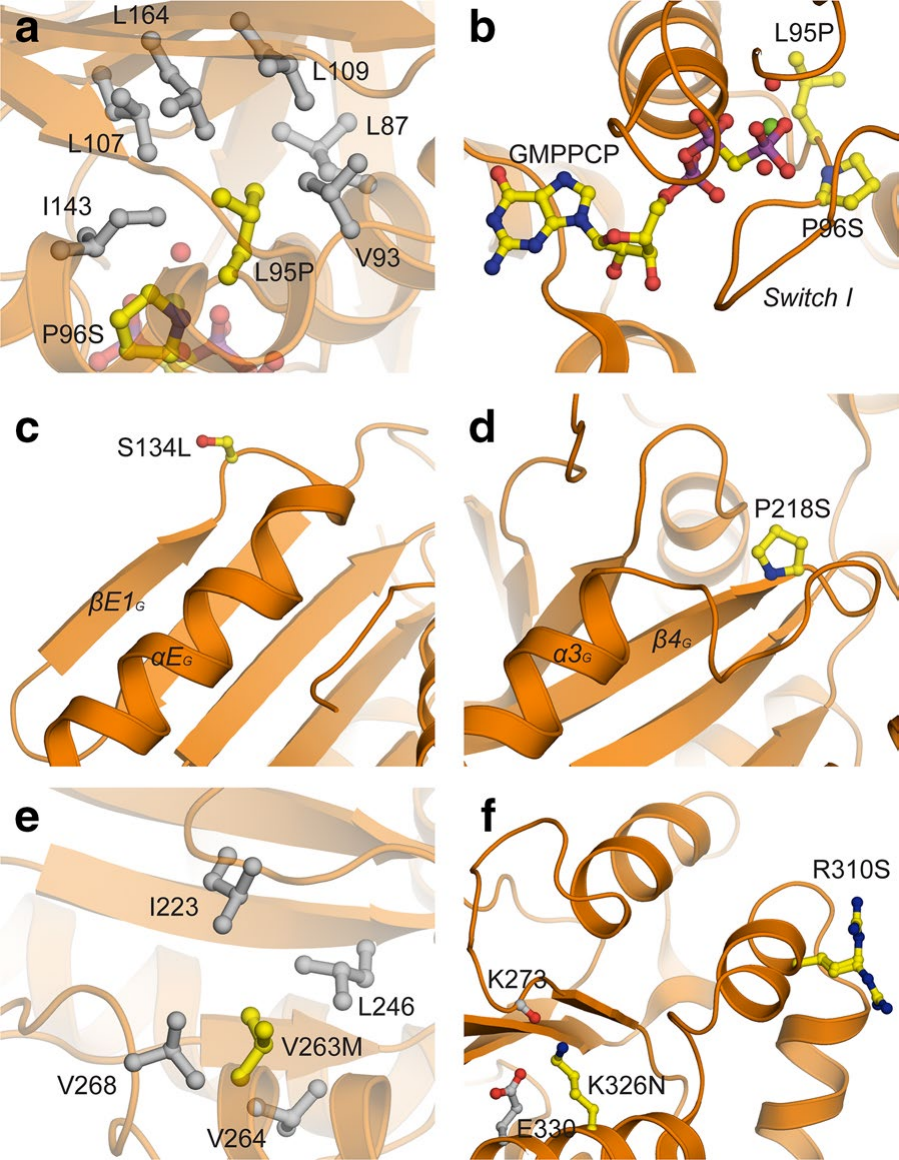
Cancer-related MxA mutations in G domain. **a** L95 is deeply buried in a hydrophobic cave. **b** P96 leads the direction of *Switch I*, which embraces guanine triphosphate nucleotide. **c** S134 is located on the surface of the G domain. **d** P218 sits at the end of β-strand 4 (*β4*_*G*_). **e** V263 is loosely enwrapped by neighboring hydrophobic residues. **f** R310 is exposed to the solvent and takes two side chain conformations, whereas K326N interacts with vicinal residues. All cancer-related mutations are shown as the original (i.e., non-mutated) residues, and the post-mutation residues are included in the labels, as L(original)95P(post-mutation), and so on. This scheme is also applied to all of the following figures

Cutaneous squamous cell carcinoma-associated single-point mutations L95P and P96S sit within *Switch I*, a key component of the active site which harbors guanine nucleotides and exists in all known GTPases [40]. L95 is deeply buried in a comprehensive hydrophobic pocket composed of L87, V93, L107, L109, I143, and L164 (Fig. 3a). Its mutation to proline is less favored in this hydrophobic network and thereby affecting the stability of the whole domain. Besides, as proline and glycine are chemically different from other amino acids in ψ and φ dihedral angles about the peptide bond, L95P mutation may lead *Switch I* to a different bending direction, and this will hinder the binding of nucleotides (Fig. 3b). Similarly, P96S mutation tends to generate more freedom for *Switch I* to swing, which is also unfavorable for nucleotide binding and hydrolysis (Fig. 3b).

S134L mutation occurs between the extra β-strand 1 (*βE1*_*G*_) and extra α-helix (*αE*_*G*_) of MxA (Fig. 3c). As the polar S134 is located on the surface of the molecule, and its side chain also protrudes outward, its mutation to hydrophobic leucine would not influence the global folding of the G domain, but just slightly change the surface entropy of the molecule.

P218S mutation emerges between β-strand 4 (*β4*_*G*_) and α-helix 3 of the G domain (*α3*_*G*_). P218 terminates *β4*_*G*_ and turns the polypeptide chain to the opposite direction (Fig. 3d). Analogous to P96S, P218S may change the original trajectory of the following loop and therefore affect the folding of the protein.

V263 is surrounded by several other hydrophobic residues, including I223, L246, V264, and V268 (Fig. 3e). Compared with the hydrophobic pocket engulfing L95, the hydrophobic environment around V263 is much less extensive. As methionine is also a nonpolar residue, V263M mutation makes no alteration of the hydrophobic property of this area. Although methionine is physically a bulkier residue than valine, there is enough space at the top of V263 to accommodate a methionine side chain. Therefore, V263M mutation would cause very limited influence to the folding and stability of the protein.

R310 sits on the surface of the molecule. The side chain of R310 points outward and is quite flexible, as two conformations can be observed in the crystal structure, which suggests that this residue is not bound by any side-chain interaction from other parts of the protein (Fig. 3f). In this situation, its mutation to a polar serine residue will neither break any intra-molecular associations nor drastically change the surface polarity of MxA. Although R310S causes the loss of some positive charge, this mutation would not negatively affect the function of the protein.

K326 is located in α-helix 5 of the G domain (*α5*_*G*_) and also has an outward side-chain conformation. It forms a hydrogen bond with the oxygen of K273 and additionally a weak salt bridge with E330 which also sits in *α5*_*G*_ (Fig. 3f). The K326 N mutation may not substantially affect these two interactions, as asparagine also has a polar side chain that can form hydrogen bonds with K273 and E330. Therefore, it is very likely that this mutation does not give rise to any major disruptions of MxA structure.

#### Single-point mutations in BSE

**Fig. 4:**
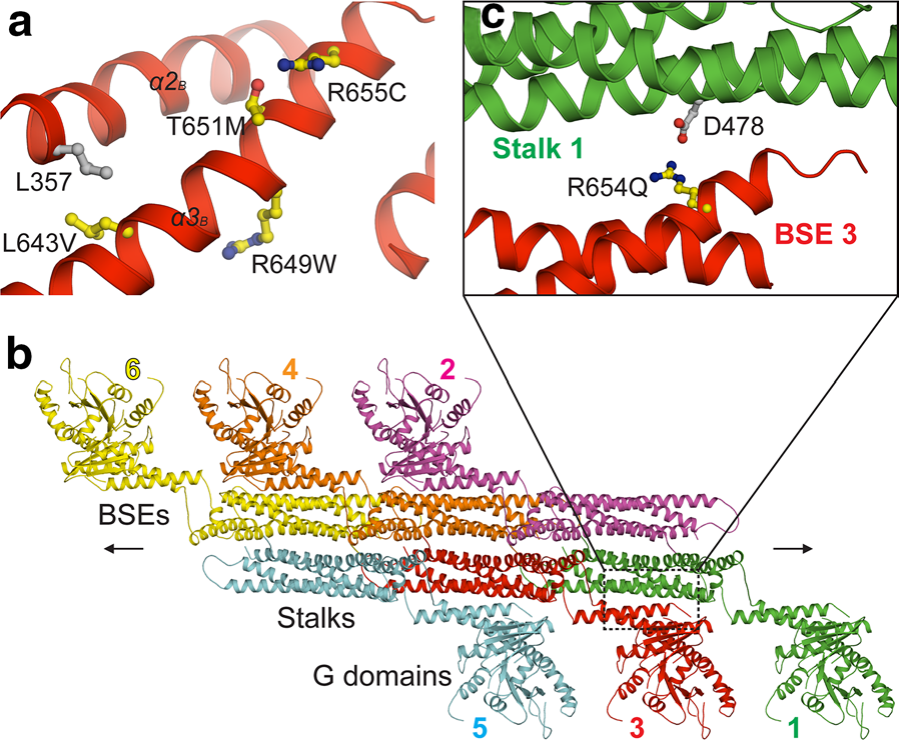
Cancer-related MxA mutations in BSE. **a** surface-located mutations and a surrounding residue. **b** the full-length MxA oligomer in the crystal lattice represented by a linear hexamer. All 6 monomers are distinguished by 6 different *colors* and indicted by *corresponding numbers*. **c** A magnified view from the *dashed rectangle* in Fig. 4b shows that the R654 is involved in inter-molecular Stalk-BSE interaction

The BSE is composed of three α-helices which are widely dispersed at the very N-terminus, middle, and the very C-terminus of MxA protein, respectively [5]. BSE is the pivot for transmitting the mechanical force generated from GTP hydrolysis at the G domain to the stalk region, so as to regulate Stalk-dependent oligomerization of the molecule [41]. Five tumor-associated single-point mutations were found in BSE and, more precisely, the α-helix 3 of BSE (*α3*_*B*_), which is also close to the end of the protein (Fig. 4a). According to the crystal structure of full-length MxA, all these 5 residues exhibit an outward side chain conformation. L643 is hydrophobically linked to L357, but its mutation to nonpolar valine would not substantially disrupt this interaction, and thus does not adversely affect the structure of the protein. The side chains of R649, T651, and R655 are all located within a spacious environment and have no contact to other elements of the molecule. Therefore, their mutations, except for mutating to glycine or proline, will lead to negligible interference in the folding of the protein. However, since arginine and threonine are both strong polar residues, whereas methionine and tryptophan possess bulky hydrophobic side chains, and cysteine is a weak-polar residue prone to various post-translational modifications, the R649W, T651M, and R655C mutations bear the possibility to interrupt the association between the protein and other protein partners or incur unexpected modifications.

In addition to its role as an intra-molecular messenger, BSE also contributes to the formation of functional homo-oligomers, which is a fundamental feature of dynamin superfamily members (Fig. 4b). R654 participates in BSE-Stalk interaction between parallel MxA monomers via a charged interaction with D478 on the other molecule (Fig. 4c). Disruption of this salt bridge by a D478A mutation results in abnormal GTPase activity and weakened oligomerization and antiviral abilities [4]. Not surprisingly, the colorectal cancer-associated R654Q mutation, which abolishes the D478-R654 salt bridge, should have a similar effect. On the other hand, glutamine still possesses the tendency to form a hydrogen bond with D478, and this can be deemed as a compensation for the loss of the salt bridge. As a result, this mutant would hardly cause negative consequence to MxA compared with the reported D478 mutant.

#### Single-point mutation in Hinge 1

**Fig. 5:**
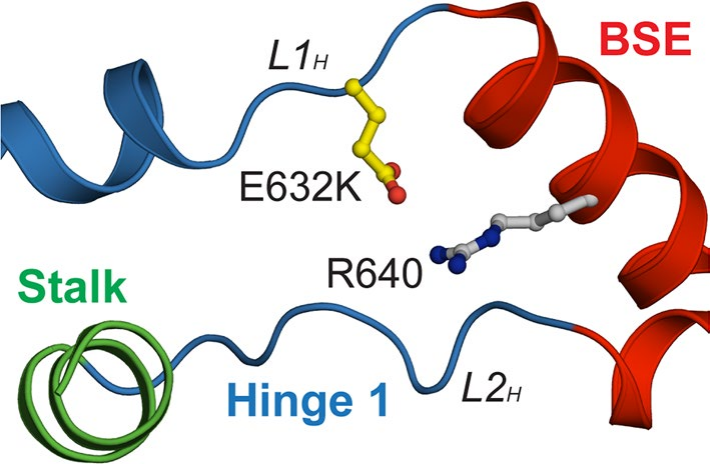
Cancer-related MxA mutations in Hinge 1. E632 in Hinge 1 forms a salt bridge with R640 to stabilize the local conformation. Part of the Stalk was removed for clarity

Hinge 1 plays a crucial role in connecting BSE and the stalk region. Extensive interactions between the two loops of Hinge 1 (*L1*_*H*_ and *L2*_*H*_) stabilize the ambient region so that the relative position of BSE and Stalk is confined from random movement [4]. The salt bridge formed by E632 on *L1*_*H*_ and R640 on *α3*_*B*_ is the determining factor in this stabilization effect (Fig. 5). Destruction of this salt bridge by mutating either E632 or R640 to alanine leads to tremendous change of GTPase activity, and almost completely abolishes the oligomerization ability and antiviral effect of MxA. Therefore, it can be well expected that the melanoma-related mutant E632K, which also disrupts the E632-R640 interaction, would be detrimental to the integrity of Hinge 1, and thus the global stability of MxA molecule.

#### Single-point mutations in the stalk region

**Fig. 6:**
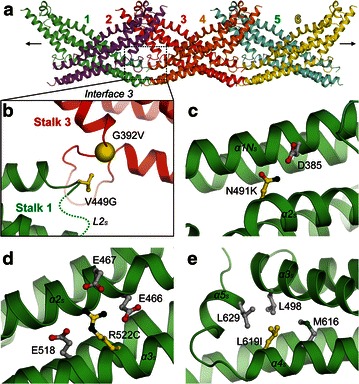
Cancer-related MxA mutations in Stalk. **a** the MxA Stalk oligomer in the crystal lattice, as represented by a linear hexamer. The monomers are *color-coded* and *labeled* in the same manner as in Fig. 4b. Note that compared with the relative direction of the full-length MxA hexamer in Fig. 4b, the Stalk hexamer has been rotated clockwise for 90° along the *X* axis, and then 180° along the *Y* axis. **b** A magnified view from the *dashed rectangle* in Fig. 6a corresponding to interface 3 shows the interaction between G392 and V449 from parallel monomers. The monomers 2 and 4 are removed for clarity. The invisible Loop 2 on the stalk (*L2*_*S*_) in this model is indicated with a *dashed line*. **c** N491 forms a hydrogen bond with D385 from another α-helix. **d** R522 is enveloped by three nearby glutamates. **e** L619 is surrounded by several hydrophobic residues

The stalk region is essential for the functional assembly of dynamin superfamily members including MxA [5, 42, 43]. In crystal lattice, MxA stalk was found forming linear oligomers via several conserved interfaces, which were then proven to also mediate the architecture of ring-like oligomers in physiological conditions [4] (Fig. 6a). Therefore, it was necessary to first determine the mutations residing in these interfaces, namely G392V and V449G. G392 is located on interface 3 and highly conserved in dynamins and Mx proteins (Fig. 6b). The G392D mutation in MxA was demonstrated to disrupt the oligomerization of the protein [4]. Moreover, its counterpart mutation (G385) in yeast dynamin leads to the breakdown of tetramer into stable dimers [44]. It is therefore not astonishing that the G392V mutation found in renal cell carcinoma deprives the oligomerization capability of MxA and thus its biological activity. In addition, the V449 residue was found to be the interaction partner of G392 on the parallel monomer in the MxA oligomer (Fig. 6b). Its mutation to glycine tends to affect the integrity of interface 3, as well as the conformation of the subsequent Loop 2 on the stalk (*L2*_*S*_). Altogether, these two mutations (G392V and V449G) are likely to impair the physiological function of MxA.

On the other hand, three additional single-point mutations that are not involved in any oligomerization interfaces have been discovered in the stalk region. N491 is a surface-located residue situated on the α-helix 2 of Stalk (*α2*_*S*_), forming a hydrogen bond with D385 on the parallel α-helix 1N-terminal part (*α1N*_*S*_) (Fig. 6c). Its mutation to positively charged arginine may strengthen this interaction, as a salt bridge can be thus introduced. Therefore, this single-point mutation would play an insignificant role in tumorigenesis or in the development of colorectal cancer [22]. Another colorectal cancer-related mutation site, R522, is also a solvent-exposed residue on *α3*_*S*_. This residue, together with three neighboring negatively charged glutamates (E466 and E467 on *α2*_*S*_, and E518 on *α3*_*S*_), constitutes a vast network of charged interactions endorsed by the salt bridges, in which R522 is at the center, to provide positive charge (Fig. 6d). It is imaginable that its mutation to weak polar cysteine causes the absence of the pivotal positive charge and lead to interference of electrostatic balance on the protein surface. In this case, the folding and stability of the whole protein would become adversely influenced. Unlike N491 and R522, L619 located on *α4*_*S*_ possesses an inward side chain conformation. It is part of a local hydrophobic core that includes L498 on *α3*_*S*_, M616 on *α4*_*S*_, and L629 on *α5*_*S*_. When mutated to isoleucine, a derivative comparable to leucine itself in both size and charge, the residue can still stably reside in and maintain this environment. As a result, this ovarian carcinoma-associated mutation L619I will lead to hardly detectable structural and functional aberrance to MxA.

#### Single-point mutations in the N′-loop and L4

The N′-loop and L4 are both intrinsically disordered loops that lack intra-molecular interactions with other residues, and therefore, it is impractical to observe them in crystal structures. The N′-loop is not conserved among Mx proteins, and its length varies among different species. This region was recently reported to be involved in the specification of viral targets [45]. Given the similar properties of threonine and serine, the T27S mutant would not chemically differ from wild-type MxA, but it is unpredictable whether this mutation would affect the protein’s interaction with viral structures. L4 is essential for membrane binding and viral resistance by direct interaction [46, 47]. However, currently only several residues in the middle of L4, but not Y538, G540, or S572, were proven to be functionally important for MxA [4, 47]. Therefore, it is difficult to predict the influence of these three mutations on MxA function, although they are not likely to disrupt the flexible conformation of L4.

## Discussion

As next-generation sequencing data of different human cancers continue to accumulate, abounding tumor-associated mutations of various types are being discovered and further analyzed. Compared with frame shift and stop codon mutations in the coding regions of given genes, it is more tricky to judge the effect of single amino acid alterations on the physiological properties of the proteins. In principal, functional proteins entail orchestrated intra-molecular interactions of the component residues and proper folding of entire polypeptide chains, which can be substantiated as so-called protein structures. In regards to analyses of single-point mutations, if the structures of the corresponding proteins have been solved by X-ray crystallography or other techniques with a decent resolution, the prediction of the effects of these mutations becomes much more reliable.

When doing such predictions, one needs to take the following points into account: the types (or more precisely, the chemical properties) of the pre- and post-mutation residues, the position of the residue, and the known inter-molecular interactions this particular protein accounts for. In our structural analysis of 22 single amino acid mutations found in various human cancers, we scrutinized all target mutants by means of the above criteria and summarized the corresponding results in Table 3. According to our judgment of how severely the structure and function of MxA would be affected, the mutations were classified into 4 groups: drastic, moderate, very moderate, and unpredictable. For drastic mutations, most of them are relevant to glycine or proline. These two residues have distinct stereochemical features compared with the other 18 amino acids. Proline has a confined configuration and usually appears at sharp turns of a peptide chain but seldom in α-helices and β-strands. Therefore, mutations either from or to proline may mislead the peptide chain to inappropriate directions and/or give rise to unexpected kinks in secondary structure elements. In contrast, glycine is the most limber residue, and thus mutations that involve this amino acid may alter the flexibility of the peptide chain at particular positions. All these outcomes are fatal to the folding of the protein.

**Table 3:** Summary of the structural analysis of cancer-related MxA single-point mutations

Mutation influence	Position in protein	Polarity before mutation	Polarity after mutation
Drastic mutations			
L95P	Buried	Nonpolar	Special
P96S	Exposed	Special	Polar
G392V	Exposed	Special	Nonpolar
V449G	Buried	Nonpolar	Special
P218S	Exposed	Special	Polar
R522C	Exposed	Positively charged	Weak polar
E632K	Buried	Negatively charged	Positively charged
Moderate mutations			
V263M	Buried	Nonpolar	Nonpolar
K326N	Exposed	Positively charged	Polar
R649W	Exposed	Positively charged	Nonpolar
T651M	Exposed	Polar	Nonpolar
T654Q	Buried	Polar	Polar
R655C	Exposed	Positively charged	Weak polar
Very moderate mutations			
S134L	Exposed	Polar	Nonpolar
R310S	Exposed	Positively charged	Polar
N491K	Exposed	Polar	Positively charged
L619I	Buried	Nonpolar	Nonpolar
L634V	Exposed	Nonpolar	Nonpolar
Unpredictable mutations			
T27S	Exposed	Polar	Polar
Y538C	Exposed	Polar	Weak polar
G540D	Exposed	Special	Negatively charged
S572Y	Exposed	Polar	Polar

According to our analysis, besides proline- and glycine-related mutations, other mutations cannot be associated with influencing levels only by single factor (position or chemical properties) (Table 3). To provide a more intuitionistic view of how these mutations may affect the structure of MxA, we performed modeling of several mutants selected from all four groups, and compared the output models to the original crystal structures. However, the calculated mutant models just bear tiny variations compared with the corresponding original structures (r. m. s. d. values less than 0.1 Å). The reason is that current structure prediction algorithms rely on the primary sequence alignment of reference structures, and differences for amino acids in stereochemistry and polarity are not taken into account during model calculation. Therefore, analyzing single-point mutations requires careful and comprehensive manual considerations in multiple aspects involving chemical properties of specific amino acid residues as well as those of the whole protein.

The seven drastic MxA mutations found in this study (L95P, P96S, G392V, V449G, P218S, R522C, and E632K) are likely to play important roles in tumorigenesis and development of corresponding human cancers, and therefore evolve to effective tumor-related biomarkers, although more biochemical and cellular assays are needed for their potential clinical applications. Moreover, it is noteworthy that all three cutaneous squamous cell carcinoma-related mutations (L95P, P96S, and P218S) lead to remarkable disruption of the protein structure, suggesting a strong association between this cancer type and MxA. Finally, our systematic approaches of structure-based analysis for single-point mutations in MxA protein are widely applicable to the evaluation of outcomes of mutations in different types of cancer for those proteins with available structural information.

## Conclusions

In this study, by analyzing 22 unique tumor-associated mutations in the human MxA protein by structural methods, we found that 7 out of 22 mutations have a high propensity to affect tumorigenesis and the development of corresponding cancers. These seven mutations are therefore more prone to potential clinical application as useful biomarkers. In addition, our research provides a good example for thorough analysis and consequence evaluation of single-point mutations on a given cancer-related protein.
